# Lipid Metabolism Reprogramming in Cancer: Insights into Tumor Cells and Immune Cells Within the Tumor Microenvironment

**DOI:** 10.3390/biomedicines13081895

**Published:** 2025-08-04

**Authors:** Rundong Liu, Chendong Wang, Zhen Tao, Guangyuan Hu

**Affiliations:** 1 Department of Oncology, Tongji Hospital, Tongji Medical College, Huazhong University of Science and Technology, Wuhan 430030, China; lrddoctor1202@126.com; 2 Hepatic Surgery Center, Tongji Hospital, Tongji Medical College, Huazhong University of Science and Technology, Wuhan 430030, China; chendongwangdoctor@163.com

**Keywords:** lipid metabolism reprogramming, tumor cells, immune cells within the tumor microenvironment, diagnosis and prognosis, therapeutic strategy

## Abstract

This review delves into the characteristics of lipid metabolism reprogramming in cancer cells and immune cells within the tumor microenvironment (TME), discussing its role in tumorigenesis and development and analyzing the value of lipid metabolism-related molecules in tumor diagnosis and prognosis. Cancer cells support their rapid growth through aerobic glycolysis and lipid metabolism reprogramming. Lipid metabolism plays distinct roles in cancer and immune cells, including energy supply, cell proliferation, angiogenesis, immune suppression, and tumor metastasis. This review focused on shared lipid metabolic enzymes and transporters, lipid metabolism-related oncogenes and non-coding RNAs (ncRNAs) involved in cancer cells, and the influence of lipid metabolism on T cells, dendritic cells (DCs), B cells, tumor associated macrophages (TAMs), tumor associated neutrophils (TANs), and natural killer cells (NKs) within TME. Additionally, the role of lipid metabolism in tumor diagnosis and prognosis was explored, and lipid metabolism-based anti-tumor treatment strategies were summarized, aiming to provide new perspectives for achieving precision medicine.

## 1. Introduction

Metabolic reprogramming is one of the most important phenotypes of tumors [1,2]. As early as 1926, Warburg discovered that cancer cells utilized aerobic glycolysis to support their rapid growth, which was different from the aerobic oxidation and anaerobic glycolysis of normal cells. This phenomenon is known as the Warburg effect [3]. Carbohydrate metabolism and lipid metabolism, two main energy supplement systems, share numerous intermediary metabolites and key enzymes, such as citrate and Acetyl coenzyme A (Acetyl-CoA) [4]. Therefore, the lipid metabolism of tumors has become the hot research topic in recent years [5]. Lipids are grouped into several categories according to their molecular features including fatty acids (FAs), phospholipids (PLs), cholesterol (CHOL), and so on [6]. The abnormal metabolism of these substances can promote the occurrence and development of tumors through cell proliferation, angiogenesis, immune suppression, cancer cell stemness, tumor metastasis [7,8,9,10].

Tumors consist of cancer cells and the tumor microenvironment (TME) [11]. Aerobic glycolysis is the traditional energy support system for cancer cells [3]. As tumors progress, they require more energy, leading to an upregulation of lipid metabolism, particularly during metastasis [12]. Due to the conservation of lipid metabolism, different tumor cells share some lipid metabolic molecules. Thus, we reviewed the shared and emerging molecules such as microRNAs (miRNAs) and long non-coding RNAs (lncRNAs). Notably, immune cells are key components of the TME [13]. As oxygen and glucose mainly flow towards tumor cells, immune cells leverage lipid metabolism as a compensatory energy support system [14,15]. The lipid metabolism reprogramming of immune cells is precise and complex [16]. To an approximate extent, lipid metabolism supports the functions of immune cells; however, excessive activation can hinder their growth, differentiation, energy supply, and anti-tumor immunity. Therefore, we provided a comprehensive insight into the molecules and signaling pathways of lipid metabolism in each type of immune cells.

Lipoproteins, including low-density lipoprotein cholesterol (LDL-C) and very low-density lipoprotein cholesterol (VLDL-C), have become widely used clinical markers in cancer management [17]. With the development of new technology, lipid metabolism-related molecules have the potential to serve as indicators for cancer diagnosis and prognosis. Tumor therapy resistance poses a significant challenge in cancer treatment [18], and lipid metabolism reprogramming offers a new insight into this problem [19,20]. Targeting lipid metabolism can improve the effectiveness of chemotherapy and immunotherapy, which is a promising therapeutic strategy [21].

Unlike previous reviews that discussed tumor lipid metabolism generally, we focused on the lipid metabolism of cancer cells and immune cells, respectively, due to their heterogeneity. We paid particular attention to the molecules and signaling pathways involved in the lipid metabolism, which can help achieve the goal of “precision medicine” [22]. Additionally, we summarized the promising molecules associated with lipid metabolism in tumor diagnosis and prognosis. Finally, we reviewed anti-tumor treatments that target lipid metabolism. We hoped that this review would deepen our understanding of the lipid metabolism reprogramming in tumorigenesis and promote the development of more efficient and precise diagnostic, monitoring, and treatment strategies.

## 2. The Overall Framework of Lipid Metabolism

**Figure 1:**
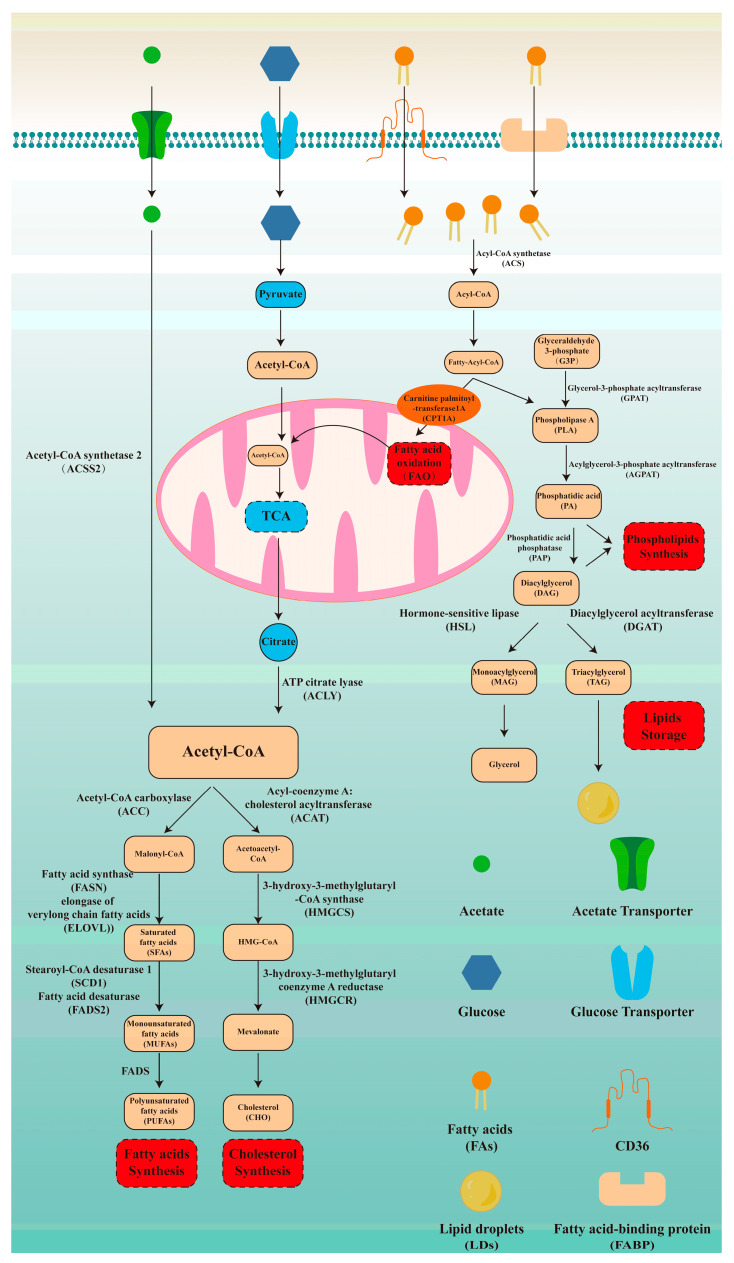
Fundamentals of lipid metabolism. Lipid metabolism is an important way to supply energy and plays a significant role in various biological processes. Therefore, understanding lipid metabolism is critical for grasping tumor lipid metabolism reprogramming. Here, we described lipid metabolism from the following five aspects: fatty acid synthesis, cholesterol synthesis, fatty acid oxidation, phospholipid synthesis, and lipid storage.

As exhibited in Figure 1, we described the lipid metabolism from the following aspects: fatty acid synthesis, cholesterol synthesis, fatty acid oxidation, phospholipid synthesis, and lipid storage. Acetyl-CoA, as the starting material for fatty acid synthesis, mainly originates from the breakdown of citrate by ATP-citrate lyase (ACLY). Alternatively, acetate bypasses the need for citrate and directly contributes to cytosolic acetyl-CoA through acetyl-CoA synthetase 2 (ACSS2) [23]. Acetyl-CoA carboxylase (ACC) catalyzes the acetyl-CoA to malonyl-CoA. Subsequently, fatty acid synthase (FASN) synthesizes palmitate by adding acetyl-CoA units to malonyl-CoA. Elongation of very long-chain fatty acids (ELOVL) extends the palmitate fatty acid chain through condensation, reduction, and dehydration. Then, saturated fatty acids (SFAs) are desaturated by stearoyl-CoA desaturase 1 (SCD1) and fatty acid desaturases2 (FADS2) to form monounsaturated fatty acids (MUFAs), which are further converted by FADS into polyunsaturated fatty acids (PUFAs). As previous research suggested, omega-3 PUFAs can suppress cardiovascular disease, tumors, and neurodegenerative diseases via relieving inflammation, while omega-6 PUFAs exacerbate them [24,25]. Therefore, we further described the above synthesis process. In the omega-3 PUFAs synthesis pathway, α-linolenic acid is converted into eicosatetraenoic acid and docosahexaenoic acid (DHA) under the catalysis of FADS2 and ELOVL. Similarly, in the omega-6 PUFAs synthesis pathway, linoleic acid (LA) undergoes desaturation mediated by FADS and elongation mediated by ELOVL to produce γ-linolenic acid and arachidonic acid. Omega-3 and omega-6 PUFAs synthesis processes are competitive as they share the same enzymes, including FADS and ELOVL. Therefore, a reasonable dietary structure is of great significance for maintaining inflammatory homeostasis.

CHOL is another important lipid that is crucial for membrane structure. Acetyl-CoA serves as the starting material for its production [26]. Initially, two molecules of acetyl-CoA condense via acyl-coenzyme A: cholesterol acyltransferase (ACAT) to form acetoacetyl-CoA. Then, 3-hydroxy-3-methylglutaryl-Coenzyme A synthase (HMGCS) catalyzes the condensation of acetyl-CoA and acetoacetyl-CoA to synthesize HMG-CoA. 3-hydroxy-3-methylglutaryl-CoA reductase (HMGCR), the rate-limiting enzyme in CHOL synthesis, reduces HMG-CoA to mevalonate (Figure 1).

Fatty acid oxidation (FAO) is an important pathway for energy supply. CD36 and fatty acid binding protein (FABP) mediate the entry of fatty acids into cells. These fatty acids are then catalyzed by ACS to generate Acyl-CoA. Carnitine palmitoyl-transferase 1A (CPT1A), as the rate-limiting enzyme of FAO, mediates the entry of acyl-CoA into mitochondria [27]. Then, once inside the mitochondrial matrix, fatty acids are oxidized by mitochondrial trifunctional protein, breaking down fatty acid chains to produce acetyl-CoA. Acetyl-CoA then enters the tricarboxylic acid cycle (TCA), which connects carbohydrate metabolism with lipid metabolism (Figure 1).

PLs are important components of lipid bilayer membranes, including phosphatidylcholine (PC), phosphatidylethanolamine (PE), phosphatidylserine (PS), and phosphatidylinositol (PI). Glyceraldehyde-3-phosphate (G3P) is converted to diacylglycerol (DAG) catalyzed by glycerol-3-phosphate acyltransferase (GPAT), acylglycerol-3-phosphate acyltransferase (AGPAT), and phosphatidic acid phosphatase (PAP). Phospholipase A (PLA), an intermediate product of the above steps, participates in the synthesis of phospholipids along with DAG. Lipid droplets (LDs), as an important form of energy storage, are also synthesized from DAG. Diacylglycerol acyltransferase (DGAT) adds fatty acyl-CoA to DAG to generate triacylglycerol (TAG). TAG is stored in the form of LDs. In addition, DAG is hydrolyzed by hormone-sensitive lipase (HSL) to produce monoacylglycerol (MAG). MAG is then hydrolyzed by MAG lipase (MGL) into the glycerol backbone and free fatty acids (Figure 1).

## 3. Lipid Metabolism Reprogramming in Cancer Cells

Different types of cancer cells share certain enzymes and transporters during lipid metabolism reprogramming, indicating universal intervention targets. Traditional cancer-related genes and pathways, such as P53, KRAS, and the mitogen-activated protein kinase (MAPK) pathway, are linked to lipid metabolism reprogramming as well. Moreover, non-coding RNAs (ncRNAs) have recently been recognized as significant regulators of lipid metabolism. In this part, we focused on three key aspects: shared enzymes and transporters, lipid metabolism reprogramming-related cancer genes, and ncRNAs, which are summarized in Figure 1 and Table 1.

### 3.1. Shared Enzymes and Transporters During the Lipid Metabolism Reprogramming

Lipid metabolism reprogramming in cancer cells primarily involves the FAO and FA synthesis, LDs formation, the mevalonate pathway-mediated CHO synthesis, and the steroid biosynthesis. Acetyl-CoA is an important hub molecule as it connects not only the lipid metabolism processes mentioned above but also glucose metabolism. To be specific, Acetyl-CoA mainly originates from ACLY-mediated citrate decomposition and ACSS2-mediated acetate decomposition, and it then participates in the FAs synthesis and the mevalonate pathway-dependent CHOL synthesis [26,64]. Consequently, cancer cells cunningly utilize acetyl-CoA-associated metabolism to reprogram lipid metabolism, aggravating the tumorigenesis [65]. FABP and CD36, two major long-chain fatty acid transporters, are upregulated in the liver, bladder, breast, and colorectal cancer [28,29,30,31]. Interestingly, FABP, in addition to its role as a binding protein for fatty acids, can also act as a binding partner for HIF-1a. Thus, FABP can bind to and activate HIF-1a to drive the lipid metabolism reprogram in hepatoma carcinoma (HCC) cells [30]. This finding was encouraging as it may partially illustrate the relationship between the hypoxic TME and elevated levels of FAs in cancer cells. The mitochondrial matrix is rich in FAO enzymes; hence, FAs must be transported from the cytoplasm to the mitochondria, and CPT1A mediates this process [27]. Breast cancer (BC) and colorectal cancer (CRC) both exhibit high expression of CPT1A [29,32]. Lin et al. found that in triple-negative breast cancer (TNBC), there was an upregulation of FAO and an increase in LDs, which was due to the two roles of CPT1A. On one hand, CPT1A, as the rate-limiting enzyme of FAO, facilitated FAO. On the other hand, CPT1A/Acyl-CoA synthetase long-chain family member 4 (ACSL4) mediated the synthesis and accumulation of LDs [32,66]. The traditional view holds that unsaturated fatty acids (UFAs) are beneficial to human health while SFAs can be harmful, leading to cardiovascular diseases and cancer [67,68,69]. However, this perspective has been challenged nowadays [70]. Generally speaking, abnormal levels of UFAs and SFAs can both contribute to tumorigenesis. Therefore, FASN and SCD1, two key enzymes of the SFAs/UFAs metabolic pathway, have attracted researchers’ interest in recent years [71,72]. Early in 2009, sterol regulatory element-binding protein 1 c (SREBP1c) was identified as a transcription factor (TF) for FASN in liver, and subsequently, the SREBP1c/FASN axis was proposed [73,74]. In subsequent studies, researchers discovered upstream regulators of the SREBP1c/FASN pathway in HCC, including CD174 and Spindlin-1 [35,36]. In TNBC, NFYAv1 upregulates the transcription of FASN to support the proliferation of triple-negative breast cancer cells [33]. Similar to FASN, SCD1 is the target gene of several lipid metabolism-related TFs, and its upstream activating molecules have been identified as well. For example, liver X receptor (LXR), an important CHOL metabolism-concerned gene, binds to the promoter region of SCD1 to mediate transcription in CRC [34]. AKT2 served as an upstream enhancer of the SREBP1-SCD1 signaling pathway, thus facilitating the progression of HCC by increasing the synthesis of MUFAs [37]. Moreover, two studies published in 2024 confirmed that M6A methylation, an emerging tumor phenotype, is involved in SCD1 in thyroid carcinoma (THCA), cervical squamous cell carcinoma, and endocervical adenocarcinoma [38,39]. Thus, interactions between FAs and CHOL synthesis are realized through transcription factor–target gene pairs such as SREBP1/FASN, SREBP1/SCD1, and LXR/SCD1. In CHOL metabolism, peroxisome proliferator-activated receptor (PPAR) and LXR are two important nuclear receptors. They have a synergistic effect on controlling intracellular CHOL levels because PPAR reduces CHOL synthesis while LXR promotes CHOL excretion [75]. This view is also applicable to cancer cells. Ju et al. reported that CD174 reprograms lipid metabolism in HCC by suppressing PPARα expression via activation of the upstream p38 MAPK signaling pathway [35]. In addition to this, LXR agonist is a novel anti-CRC strategy targeting CHOL metabolism [34]. As the steroid biosynthesis process is closely related to CHOL, reprogramming steroid metabolism in tumor cells plays a critical role in numerous tumors, including endometrial carcinoma, prostate cancer, ovarian cancer, testicular cancer, adrenal cortical carcinoma, leukemia, lymphoma, lung cancer, and BC [76,77,78,79]. Cytochrome P450 family 11 subfamily A member 1 (CYP11A1) plays an important role in the recurrence and metastasis of prostate cancer. Xu et al. reported that CYP11A1 mediated the recurrence of castration-resistant prostate cancer by enhancing the production of dihydrotestosterone and activating androgen receptor (AR) signaling [46]. Subsequent studies found that prostate cancer tumor cells expressing CYP11A1 can produce pregnenolone, promoting bone metastasis through prolyl 4-hydroxylase subunit beta (P4HB). This finding also applies to bone metastasis in BC and melanoma [41]. Interestingly, the intracellular localization of CYP11A1 is also important. In gastric cancer (GC), the interaction between CYP11A1 and cytotoxin-associated gene A redistributed mitochondrial CYP11A1 to the outside of the mitochondria. This relocation then led to CHOL accumulation in mitochondria and ultimately promoted the proliferation of GC cells. Helicobacter pylori could exacerbate the above process [40]. Furthermore, cytochrome P450 family 11 subfamily B member 1 (CYP11B1), cytochrome P450 family 11 subfamily B member 2 (CYP11B2), and cytochrome P450 family 17 (CYP17), contribute to steroid hormone-mediated tumorigenesis. For instance, CYP17 has become a recognized therapeutic target for prostate cancer treatment, and recent studies suggest it is a promising therapeutic target for breast cancer and neuroblastoma [47]. Specifically speaking, seviteronel (a novel CYP17 inhibitor and AR antagonist) can enhance the sensitivity of AR-positive triple-negative breast cancer cells to radiotherapy with low side effects [42,43]. In addition, the overactivation of the AR-SCAP-SREBPs-CYP17/HMGCR axis in tumor cells contributes to the proliferation and migration of neuroblastoma, whereas abiraterone acetate combined with statins could reverse these phenomena [50]. CYP11B1 and CYP11B2, as key enzymes of adrenal corticosteroids, mediate the development of adrenal cortical tumors and prostate cancer [48,51]. Hydroxysteroid dehydrogenases promote the growth of prostate cancer, breast cancer, and colorectal cancer. In prostate cancer, 3b-hydroxysteroid dehydrogenase type 1 (3βHSD1) acted as a potential driver of treatment resistance [49,80]. Similarly, 3βHSD1 was a key mediator of endocrine resistance in ER+ breast cancer, and liver receptor homolog-1 (LRH1) enhanced this resistance [44]. Furthermore, 11b-hydroxysteroid dehydrogenase type 2 (11βHSD2) promoted colorectal cancer cell migration, invasion, and metastasis through the FGFBP1/AKT pathway [45]. In conclusion, the interactions between specific lipid metabolism enzymes and transporters are prevalent across various types of cancer, highlighting their potential significance in cancer biology. Therefore, targeting the above shared enzymes and transporters is promising for cancer prevention and treatment.

### 3.2. Lipid Metabolism Reprogramming-Related Cancer Genes

Cancer genes can be categorized into oncogenes and anti-oncogenes, which influence cancer cell characteristics such as proliferation, cell cycle, apoptosis, and metabolic reprogramming [81,82]. Previous studies have primarily focused on glucose metabolism, which is closely linked to lipid metabolism; therefore, we reviewed their role in lipid metabolism reprogramming in this part [83]. P53, a well-known anti-oncogene, could suppress tumorigenesis through lipid metabolism [84]. A 2024 study confirmed this, as researchers found that exonuclease 1-induced *P53* inhibition promoted the expression of SREBP1, thereby enhancing FAs synthesis to provide enough energy for the prostate cancer cell proliferation and invasion [52]. Besides, *P53* also connects tumors with metabolic diseases such as atherogenesis, suggesting that it could serve as a comprehensive therapeutic target [84,85]. It is worth noting that, although *P63* belongs to the *p53* family, it behaves as an oncogene in squamous cell carcinomas (SCC) instead of an anti-oncogene. Specifically speaking, the SREBF1/TP63/Kruppel-like factor 5 (KLF5) loop mediated the synthesis of FAs, sphingolipids (SLs), and glycerophospholipids (GPLs) while also regulating the transcription of genes within mTOR signaling pathways. The above process ultimately facilitates the proliferation and invasion of cancer cells [53]. Classic cancer-related pathways like PI3k/AKT and MAPK are composed of cancer genes and can mediate lipid metabolism as well. The PI3k/AKT pathway, which is upstream of SREBP1, plays an important role in HCC. As we described in Shared Enzymes and Transporters during the lipid metabolism Reprogramming, the CD174/AKT/mTOR/SREBP1c/FASN, ACC1 cascade leads to the disorder of FAs synthesis [35]. In a 2024 study, Tu et al. illustrated a more detailed mechanism. The PD-L1/EGFR/ITGB4 complex in the cell membrane activated the PI3K/mTOR/SREBP-1c signaling pathway, which promoted the reprogramming of lipid metabolism in cancer cells [55]. In addition to HCC, the relationship between PI3k/AKT/mTOR pathway and lipid metabolism reprogramming was also found in GC [54]. The MAPK pathway is categorized into four sub-pathways, namely, (a) the classical MAPK pathway (RAS/RAF/MERK-1(2)/ERK-1(2) pathway), (b) the p38 pathway, (c) the JNK pathway, and (d) the ERK5 pathway. P38, KRAS, MEK5, and ERK5 are biomarkers within these pathways [86]. For instance, the downregulation of FAO in HCC resulted from the CD174/p38 /PPARα axis [35]. KRAS, the biomarker of the classic MAPK pathway, promoted LDs accumulation through HSL, thus fueling the metastasis of pancreatic cancer cells [58]. The classical MAPK pathway has become a traditional therapeutic target for lung cancer [57]. Likewise, Cristea et al. revealed MEK-5/ERK-5 as a new target for small-cell lung cancer (SCLC) from the perspective of lipid metabolism [56]. In summary, the aforementioned cancer genes either directly mediate lipid metabolism or interact with lipid metabolism genes. Thus, lipid metabolic reprogramming offers a new perspective on the role of cancer genes in the pathophysiological processes of cancer cells.

### 3.3. Lipid Metabolism Reprogramming-Related Non-Coding RNAs (NcRNAs)

NcRNAs consist of miRNAs, lncRNAs, and circRNAs (circular RNAs), with circRNAs being a specific type of lncRNAs [87,88,89]. MiRNAs bind to the 3′ untranslated region (3′UTR) of messenger RNAs (mRNAs) and then downregulate the target gene levels, primarily by promoting mRNA degradation, reducing mRNA stability, and inhibiting translation [87]. Therefore, miRNAs can reprogram lipid metabolism in this way as well. In lung cancer, miR-15a-5p blocked the expression of ACSS2, ACC, and FASN in two ways. On one hand, miR-15a-5p directly degraded ACSS2 mRNA by attaching to its 3′UTR, and on the other hand, miR-15a-5p suppressed the transcription of ACC and FASN by reducing histone H4 acetylation levels. These processes repressed FA synthesis, finally inhibiting lung cancer cell proliferation, invasion, and migration [59]. Arachidonic acid, an omega-6 PUFA, plays major roles in immune response, inflammation, cell proliferation, and differentiation rather than energy supply [90,91]. Fatty acid amide hydrolase (FAAH), which hydrolyzes arachidonoyl ethanolamide (AEA) into arachidonic acid and ethanolamine, has similar functions [92]. From the perspective of arachidonic acid metabolism, Yang et al. illustrated that FAAH promoted GC progression via the AEA/LPA/COX-2/PGE-2 axis while miR-1275 was able to block this process by binding to FAAH [60]. The competing endogenous RNAs (ceRNAs) mechanism, in which miRNAs competitively bind to target gene mRNAs with lncRNAs, has been widely validated in multiple biological processes, such as proliferation, invasion, metastasis, and metabolism reprogramming, across different types of cancers [93,94,95]. In recent years, lipid metabolism has been found to be involved in this process. For example, lncRNA lnc030 maintained the stability of squalene epoxidase (SQLE) mRNA and enhanced the synthesis of CHO in BC [61]. LncRNA SLC25A21-AS1, a lipid metabolism-related lncRNA, promoted the esophageal squamous cell carcinoma (ESCC) cells’ migration and proliferation via the SLC25A21/NPM-1/c-Myc axis [62]. A 2024 study explored the extracellular vesicle transportation in lung cancer. LncRNA ROLLCSC entered lung cancer cells with the aid of vesicles and then promoted lung cancer metastasis by triggering triglycerides (TGs) and free fatty acid accumulation through competing with miR-5623-3p and miR-217-5p for lipid metabolism-related genes, including Tnpo1, Npr3, Bace2, and Sema5a [63]. Generally speaking, lipid metabolism within cancer cells could be repressed primarily via two approaches: (a) activating lipid metabolism-related miRNAs; (b) inhibiting lncRNAs involved in lipid metabolism. Considering the great number and complex interactions of ncRNAs, more studies are necessary.

## 4. Lipid Metabolism of Immune Cells in Tumor Microenvironment

**Figure 2:**
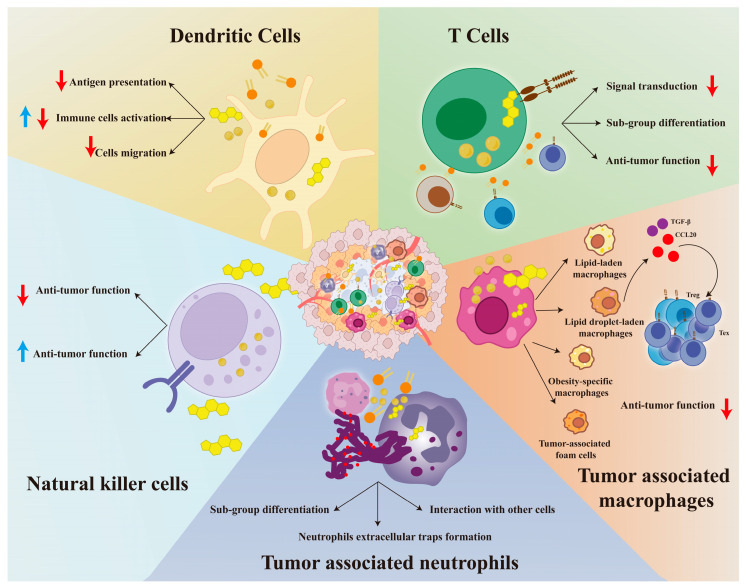
The landscape of immune cells with lipid metabolism reprogramming in the tumor microenvironment. We demonstrate how lipid metabolism affects DCs, T cells, NKs, TAMs, and TANs in Figure 2. For DCs, we focus on the effects of lipids on their antigen presentation, activation, and migration. For T cells, we show the influence of lipids on their differentiation, signal transduction, and anti-tumor immune response. For NKs, we have found that lipids have a dual effect on their anti-tumor immunity. For TANs, we focus on changes in their cell differentiation, neutrophil extracellular traps formation, and cell interactions. For TAMs, we have identified four high-fat subgroups and found that they recruit Treg and Tex cells by secreting TGF-β and CCL-20, thereby creating an immunosuppressive microenvironment. Further details are provided in Section 4. Abbreviations: DCs, dendritic cells; NKs, natural killer cells; TAMs, tumor-associated macrophages; TANs, tumor-associated neutrophils. Red ↓: indicates inhibition; Blue ↑: indicates activation.

In the tumor microenvironment (TME), immune cells mainly include dendritic cells (DCs), T cells, B cells, tumor-associated macrophages (TAMs), tumor-associated neutrophils (TANs), and natural killer cells (NKs) [13,96]. Unlike cancer cells, in addition to being an energy supplement, lipid metabolism reprogramming plays more complicated roles, including signal transduction, subgroup differentiation, and anti-tumor immunity. Lipid metabolism exerts a complex influence on the function of immune cells, regarding the differences among different types of immune cells [16]. Therefore, it was worthwhile to review the lipid metabolism of immune cells within the TME, and we present them in Figure 2 and Table 2.

### 4.1. DCs

DCs are the most effective antigen-presenting cells (APCs) that present tumor antigens and then provide T cell co-stimulatory activation signals [13]. Thus, DCs are always deemed to be the trigger point of the tumor immune response. Lipid metabolism is essential for the development, maturation, differentiation, and immune function of DCs. 12/15-lipoxygenase (12/15-LOX)-mediated enzymatic lipid oxidation is representative as it can regulate the inflammatory response of monocytes and the maturation of DCs [121,122]. It has been widely accepted that the accumulation of LDs would impair the normal functions of DCs [123]. In mesothelioma (MESA), plasmacytoid DCs with high lipid levels show low immune response capacity, including antigen presentation, migration, and T cell activation during tumor progression [99]. Next, researchers explored the mechanism of lipid accumulation and found that mesothelioma cell-derived TGF2-β2 overactivated the diacylglycerol acyltransferase [124]. In the radiation-induced thymic lymphoma model, Gao et al. observed an upregulation of serum LPL, FABP4, TAG, and lipid accumulation in DCs, accompanied by low expression of co-stimulatory molecules (CD80, CD86, Ia, CD40, and CCR7) and cytokines (IL-12 p40, IL-1, and IFN-γ), making DCs unable to stimulate T cells effectively [97]. This finding was significant as it not only proposed a new mechanism for radiation-induced cancer but also provided new insights into radiotherapy-induced immunosuppression. However, Ibrahim et al. reported contrary results. In liver DCs, high lipid concentrations correlated with high immunogenic characteristics, including the activation of T cells, NK cells, and NK-T cells [125]. This interesting phenomenon indicated that maintaining an appropriate lipid level was crucial for the normal function of DCs. In addition, organ heterogeneity has a significant impact on lipid metabolism and immune function in DCs. As the liver is an important fat storage organ, DCs may adapt to the high lipid environment. Therefore, DCs can maintain pro-inflammatory immune function with a high concentration of lipid. For instance, DCs promoted lipid synthesis through the activation of acetyl-CoA carboxylase, which contributed to the immune response in liver injury and fibrosis [126]. Since immunosuppression is a key feature of tumors, DCs with high lipid concentration may improve the prognosis of HCC patients. Although Cheng et al. have constructed the pan-cancer cell map of DCs, research on the lipid metabolism differences in DCs among different tumors remains insufficient [127]. This indicates a clear direction for future research. DCs are also the victim of overactivated FA metabolism and CHOL metabolism. For instance, in a mouse model with B16 F10 melanoma cells, FABP5, the fatty acid transporter, reprogrammed lipid metabolism in DCs and promoted the generation of FOXP3^+^ regulatory T cells, building an immunosuppressive TME [98]. This can be attributed to the FAs’ overburden. On one hand, FAs are involved in the synthesis of TGs, leading to an excess of LD storage. On the other hand, FAs participate in FAO, producing a large amount of reactive oxygen species (ROS) that can damage cellular membranes and organelles. This view is supported by a high-fat diet model [128]. Therefore, inhibiting the endogenous FAs synthesis and exogenous FAs uptake may be a strategy to restore the immune response of DCs. TPOP, a newly developed in situ nano vaccine, exploited this to suppress CRC and melanoma [129]. Likewise, LXR antagonist PFM037 restored the DCs’ differentiation in TME by reversing the CHOL transport pathway [130].

### 4.2. T Cells

Lipid metabolism reprogramming primarily influences T cells based on three aspects: dynamic energy supply, signal transduction and membrane molecules, and sub-group differentiation and anti-tumor function.

#### 4.2.1. Dynamic Energy Supply

The energy supply system is dynamic, involving lipid metabolism and glycolysis throughout the T cell differentiation and development. Lipid metabolism is more active in resting T cells and memory T cells (T_mem_) due to the high energy reserves. However, in effector T cells, glycolysis, which is the fastest method for energy supply, replaces lipid metabolism as the main source of energy to support rapid immune responses [131]. Interestingly, although the pentose phosphate pathway is not a main energy supply mechanism, it is upregulated alongside FAO in T_mem_. The underlying mechanism involves the pentose phosphate pathway generating glutathione, thus protecting cells from the oxidative stress induced by FAO [132].

#### 4.2.2. Signal Transduction and Membrane Molecule

The T cell receptor (TCR) is composed of TCRα chains, TCRβ/TCRγ chains, TCRδ chains, and the CD3 complex that includes CD3ε, CD3δ, and CD3γ chains. TCR-CD3 complex supports the normal function of T cells, and CD28 serves as the co-stimulator [133,134]. TCR-CD3-mediated TCR signal transduction depends on lipid rafts, with CHOL playing an indispensable role [135]. In 2012, Molnár et al. discovered that the TCRβ chain served as the binding site for CHOL and reported a CHOL/TCRβ/CD3ε signal transduction pathway [136]. In the following period, several CHOL-related metabolites have been found to affect the signaling transduction of T cells in this way. CHOL sulfate, a cancer-derived CHOL analogue, can block TCR signaling through two mechanisms: (i) CS binds competitively to the TCRβ chain, causing CD3γ to adopt an inactive conformation [137,138]. (ii) The negatively charged CS binds to the positively charged CD3ε, thereby preventing CD3ε release [100]. Spermidine, another cancer cell-secreted metabolite, reduces CHOL levels by suppressing the expression of genes involved in CHOL biosynthesis, such as HMGCR and SQLE, as well as CHOL uptake genes like the low-density lipoprotein receptor. This reduction ultimately hinders TCR aggregation [101].

#### 4.2.3. Subgroup Differentiation and Anti-Tumor Function

T-bet is a crucial TF for the differentiation of Th1 cells, and IFN-γ is a significant cytokine associated with anti-tumor activity [139]. The phosphatidylinositol-4, 5-diphosphate (PIP2)-related metabolism plays an indispensable role in the differentiation and function of CD4^+^ Th1 cells. To be specific, the PIP2/PLC-γ/DAG/PKC pathway and the PIP2/PI3K/ PIP3/AKT/mTOR GSK pathway both activate and stabilize T-bet. Subsequently, T-bet attaches to the promoters of the IFN-γ and IL-12R genes to mediate transcription, thereby promoting the differentiation and function of Th1 cells [140,141]. Besides, SCD regulates interactions among T cells. In CD4^+^ T cells, higher SCD expression increases Th1 cell markers such as T-box transcription factor 21, IL-2, and IFN-γ. At the same time, it decreases the markers of Tregs, including FOXP3 and TGF-β. Moreover, SCD increases the secretion of CXCL11 from CD4^+^ T cells, and the binding of CXCL11 to CXCR3 on CD8^+^ T cells subsequently boosts their cytotoxic activity [102]. The research has demonstrated that LA is essential for the anti-tumor activity of CD8^+^ T cells across different cancer types [103]. In the following year, Ma and colleagues further investigated the underlying mechanisms and suggested that HCC tumor cells upregulated the uptake of LA through the LINC01116/EWS RNA-binding protein 1 (EWSR1)/PPARA/FABP1 axis [142]. The researchers demonstrated that in CRC, tumor-associated foam cells suppressed the anti-tumor immune response via TGF-β-related pathways, leading to an increase in T cell exhaustion and an enrichment of Tregs [143]. Subsequently, Huang et al. discovered that increased ATP6V0A1-dependent CHOL uptake in tumor cells may explain this phenomenon [144]. As the liver plays a crucial role in lipid metabolism and immune function, liver cancer is linked to immune cell activity and lipid metabolism disorders as well. For example, in HBV-associated liver cancer, the accumulation of long-chain acylcarnitine led to T cell exhaustion and functional defects [104]. Similarly, the S100 calcium-binding protein A10 (S100A10) activated the cPLA2 and 5-lysyl oxidase (5-LOX), initiating lipid metabolic reprogramming and upregulating LTB4 levels. This process promoted the exhaustion of CD8^+^ T cells and the immune evasion of HCC cells, which ultimately facilitated the growth and migration of cancer cells [105].

### 4.3. TAMs

TAMs are primarily categorized into two subsets, namely, M1 and M2, which mediate anti-tumor and pro-tumor mechanisms, respectively [145]. Lipid metabolism, including FAs uptake, FAs utilization, and CHOL expulsion, has been found to play an important role in the above polarization process [146]. Apart from M1 and M2 subsets, recent studies have identified a series of lipid metabolism programming-related TAM subsets, such as lipid-laden macrophages (LLMs), tumor-associated foam cells (TAFs), obesity-specific macrophages (OSMs), and lipid droplet-laden macrophages (LDLMs). All of them exhibited a pro-tumoral phenotype. For example, after engulfing CHOL-rich myelin debris, TAMs turned into LLMs and then transferred myelin-derived lipids to glioblastoma (GBM) cancer cells via an LXR/Abca1-dependent way, thereby supporting the metabolic demands of mesenchymal GBM [106]. Similar results have been reported in pancreatic cancer [107]. TAFs, a type of lipid droplet-loaded macrophage, established a pro-tumor microenvironment (hypoxia, mesenchymal transition, angiogenesis) and impaired phagocytic ability, which together promoted GBM tumorigenesis [108]. This finding partly aligns with a study conducted on orthotopic GBM models in male mice [109]. In OSMs, nuclear receptor subfamily 1 group H member 3 acted as a TF and regulated FABP4 expression by interacting with the DNA of SREBP1, eventually increasing the proliferation of BC tumor cells [110]. In LDLMs, LDs extended the survival of LDLMs and promoted the secretion of CCL20, recruiting CCR6^+^ Tregs to HCC tissue, which helped build a pro-tumor microenvironment [111]. Interestingly, lipid metabolism exerts an influence on the origin of TAMs. As Qin et al. demonstrated, CD36 expanded the population of circulating derived monocytes while decreasing the tissue-resident-derived monocytes through CCL2/CCR2/p110γ signaling [147]. Given the importance of lipid metabolism reprogramming within TAMs, targeting lipid metabolism molecules of TAMs rather than the whole body would yield greater benefits. Correspondingly, therapeutic targets such as scavenger receptors and ApoA1 have been proposed [109,148].

### 4.4. TANs

Lipid metabolism plays a critical role in neutrophil development, anti-infection ability, and anti-tumor capacity [149,150]. Similar to TAMs, TANs are deemed as a double-edged sword in the anti-tumor process. To be specific, TAN1 exhibits an anti-tumor phenotype, whereas TAN2 has a pro-tumor phenotype. Early in 2009, Fridlender et al. revealed that TGF-β, an immune-suppressive cytokine, caused TANs to differentiate into TAN2 [151]. Lipid metabolism mediates TAN polarization as well. For example, acrolein, a type of lipid peroxidation byproduct in a hypoxic environment, induced TANs to differentiate into the TAN2 phenotype by reacting with Cys310 of AKT and suppressing AKT activity [112]. Neutrophil extracellular traps (NETs), composed of neutrophil granule proteins and DNA strands, play a dual role in tumorigenesis. On one hand, with MMP-9, cathepsin G, and neutrophil elastase (NE), NETs could induce tumors via promoting tumor distant site spread, shielding immune cell infiltration, killing immune cells, and provoking tumor angiogenesis and cancer-associated thrombosis [152,153]. On the other hand, NETs suppress tumors by killing cancer cells and stimulating the immune system [152]. Recent studies have found that lipid metabolism reprogramming could influence NETs and, thereby, affect their anti-tumor ability. For recurrent GBM, acyl sphingosine amidohydrolase 1 (ASAH1, an enzyme that regulates ceramide metabolism) increases alongside MMP-9 within NETs, which promotes the formation of NETs and their pro-tumor function [113]. In CRC, enoyl-CoA δ-isomerase 2 (ECL2) reduces ether-lipid-mediated IL-8 expression in CRC cells by repressing the peroxisomal localization of alkylglycerone phosphate synthase, thereby suppressing the neutrophil recruitment and NETs formation, which suggests the anti-tumor role of ECL2 [114]. TANs have a complicated interaction with immune cells and tumor cells. In CRC, the CHOL intake and lipid raft-mediated anti-tumor signaling of NK cells are both disturbed by CD16^+^ TANs. In the mechanism, the CD16 /TAK 1/NF-κB axis of CD16^+^ TAN is activated, upregulating CD36 and LRP1, which finally leads to the over-accumulation of CHOL [115]. The influence of TANs on tumor cells is bidirectional, and lipid metabolism can regulate this process. On one hand, TANs provide lipids for cancer cells, satisfying the energy needs of tumorigenesis. As demonstrated during the adeno-to-squamous transition (AST) of lung cancer, TANs take up TG through micropinocytosis and then transfer lipids into cancer cells to support the proliferation and AST. The above process is mediated by TET2-STAT3-CXCL5 [116]. On the other hand, cancer cells release lipid metabolites to induce TANs into an immunosuppressive phenotype. Platelet-activation factor (PAF), a common tumor-derived lipid metabolite, is an example. In cancer cells, PAF promotes TANs to differentiate into an immunosuppressive phenotype called T3, which represses the anti-tumor toxicity of CD8^+^ T cells [117,154]. Notably, T3 is defined as the terminal differentiation form of TANs, located in the hypoxic and glycolytic tumor niche, with a high angiogenic capacity. At the molecular level, T3 expresses high levels of dcTRAIL-R1, VEGFA, THBS1, LGALS3 and HK2 [154]. This PAF-mediated immunosuppression of TANs has been observed in numerous tumors, such as pancreatic ductal adenocarcinoma (PDAC), CRC, lung cancer, and ovarian cancer [117]. Therefore, PAF has been recognized as a promising anti-tumor immunotherapy target [155].

### 4.5. NKs

As previously recognized, the over-accumulation of lipid within NKs would impair anti-tumor immunity. For example, during the postoperative period of CRC patients, the upregulation of CD36 led to increased lipid content in NK cells, decreased production of granzyme B and perforin, and suppression of anti-tumor cytotoxicity [118]. In the following years, Jiao et al. took a deep insight into the underlying mechanism. They demonstrated that lipid accumulation impaired the anti-tumor immunity of NKs persistently, as it blunted P300-mediated c-myc acetylation and shortened its protein half-life in NK cells, which, in turn, reduced P300 accumulation and H3K27 acetylation [119]. However, this perspective has recently been challenged. In 2024, a study based on a mouse liver cancer model reported the opposite results. In the high-CHOL diet group, NKs increased the expression of NK cell-activating receptors (natural cytotoxicity triggering receptor 1 and natural killer group 2, member D), effector function makers (granzyme B and perforin), and cytokines and chemokines, alongside a high level of CHOL [120]. The differences in lipid composition and metabolism processes may be reasonable explanations for the different anti-tumor immunity of NKs under lipid accumulation. CHOL, as an important component of cell membranes, plays a crucial role in maintaining the immune function and survival of NKs. As early as 2007, studies found that statins inhibited NKs’ cytotoxicity and degranulation by depleting membrane rafts [156,157]. Subsequently, Zhang et al. discovered that in CRC, depriving CHOL intake in NKs disrupted the formation of lipid rafts and blocked their anti-tumor signaling [115]. However, excessive CHOL can inhibit NK function by impairing lipid rafts and inhibiting cytokine production [158]. Therefore, maintaining appropriate CHOL levels is essential for the anti-tumor immunity of NKs [159]. FAO plays an important role in maintaining the proliferation and immune function of NKs [160]. Cyclic fasting could improve the NK-mediated immunity against tumors by activating FAO [161]. It is worth noting that excessive storage of FA can inhibit mTORC1 and damage glucose metabolism, which impairs the anti-tumor immunity of NKs [162,163,164]. Therefore, FAO, rather than FA accumulation, promotes NK cell anti-tumor immunity. In addition, PC, as an immunosuppressive metabolite, disrupted the membrane order and impaired cytotoxicity of NKs in high-grade serous ovarian cancer [165]. Since lipid-mediated effects on NKs occur at specific times, focusing on lipid exposure duration could be a promising direction for future studies.

## 5. The Influence of Lipid Metabolism Genes and Metabolites on the Diagnosis and Prognosis of Tumors

Lipid metabolites are highly effective for diagnosing and predicting the outcomes of cardiovascular diseases [166], kidney diseases [167], and diabetes [168]. In 2019, Islam et al. proposed trans fatty acids as a serious risk factor for cancers [169]. With the rapid development of multi-omics and next-generation sequencing technology, transcriptomics and metabolomics shed light on the lipid metabolism indicators associated with cancer. Therefore, in this section, our review is based on the following three aspects: (i) genes and metabolites related to lipid metabolism; (ii) prognostic models based on lipid metabolism; (iii) advantages over conventional lipid metabolism markers.

### 5.1. Genes and Metabolites Related to Lipid Metabolism

For BC, lipid metabolism-related genes, including *CD36*, *AQP7*, *LIPE*, and *AKR1C1* were significantly upregulated in susceptible mammary epithelium with Log_2_ (Fold Change) > 1.5 and *p*-value < 0.05. Meanwhile, PPAR (PPARα, PPARγ) served as a key upstream regulator of these genes in the susceptible stroma. Therefore, elevated levels of *CD36*, *AQP7*, *LIPE*, and *AKR1C1* in mammary epithelial cells may serve as promising indicators for identifying high-risk breast cancer groups [28]. Drug resistance is the main reason for the poor prognosis of BC patients. As a recent study illustrated, *CD36* mediated drug resistance by supplying energy for epithelial–mesenchymal transition through increased FA uptake. Therefore, *CD36* is an important indicator of trastuzumab resistance in human epidermal growth factor receptor-2 (HER2)-positive BC [170]. Similarly, *AKR1C1* was associated with shorter recurrence-free survival and alpelisib resistance in HER2-positive BC patients [171]. The above phenomenon can be partially attributed to the disruption of the immune microenvironment [171,172]. Lipid metabolites are also crucial for tumor diagnosis and prognosis. As early as 2011, Lee et al. proposed the hypothesis of classifying non-small-cell lung cancer (NSCLC) based on lipid profiles [173]. Subsequent studies further confirmed that lipid metabolites played an important role in the diagnosis and prognosis of NSCLC. For instance, serum PC and lysoPC were identified as biomarkers for NSCLC diagnosis [174,175]. In addition, a retrospective study based on 551 patients suggested that preoperative high serum TGs indicated shorter overall survival for NSCLC [176]. In 2022, Wang et al. further identified lysoPC, PC, and TGs as important indicators for the early NSCLC detection using single-cell sequencing and lipidomics. They also confirmed the high sensitivity and specificity of these markers in a large sample cohort study [177]. In subsequent years, the value of plasma lipid levels in the diagnosis, prognosis, immune filtration, and immunotherapy response was confirmed in various tumors, including BC, CRC, and PDAC [178,179,180,181].

### 5.2. Prognostic Models Based on Lipid Metabolism

In recent years, prognostic models based on lipid metabolism and tumor immunity have been established in BC and GC. For instance, in BC, Shen et al. established a prognostic model based on the following genes: *HIBCH*, *OSBPL10*, *FIG4*, *OCRL*, *CPT1A*, *INPP5F*, *PTGES3*, *HSP90AA 1*, and *ALOX15* with the weight coefficient at 0.2317, 0.2823, 0.1183, 0.2407, 0.1499, 0.0423, 0.2273, 0.0177, and 0.1636, respectively. Then, they divided BC patients into high- and low-risk groups [182]. For GC, researchers built a prognostic model based on *APOA1*, *BCHE*, *CYP19A1*, *PLA1A*, and *STARD5*. The high-risk group experiences significantly lower overall survival compared to the low-risk group (*p* < 0.001) [183]. In terms of the mechanism, the high-risk group was positively correlated with M2 macrophages in both BC and GC, indicating the connection between lipid metabolism and tumor immunity [182,183]. Therefore, constructing prognostic models related to lipid metabolism and immunity is the direction for future tumor diagnosis and prognosis.

### 5.3. Advantages and Challenges for Lipid Metabolism Indicators

The lipid metabolism profile of blood and malignant effusion, as a non-invasive examination, can provide important information for the diagnosis and prognosis of tumors. Since the biopsy-induced tumor cell implantation was common in various tumors, our inspection method is meaningful [184,185,186,187,188]. As we described in Genes and Metabolites Related to Lipid Metabolism, PC, lysoPC, TG, and LDL were associated with unfavorable prognosis for NSCLC, BC, CRC, and PDAC. Blood lipid profiles could improve prognostic value when accompanied by traditional indicators. For example, a prognosis model based on TC, HDL-C, TG, and Ki67 has better prognostic predictive ability than Ki67 alone in advanced-stage small-cell lung cancer [189]. Furthermore, establishing a prognosis model based on lipid metabolism-related genes could improve the diagnostic efficacy. As Liu et al. found, a prognostic model based on *ADH1C*, *APOE*, *RAP1GAP*, *NPC1L1*, *P4HB*, *SOD2*, and *TNFSF10* served as an independent risk factor for PDAC. This model was superior to the clinical stage, regardless of the Cancer Genome Atlas training set or the International Cancer Genome [190].

Several challenges must be addressed when applying lipid metabolism indicators in clinical practice. Firstly, since blood lipid levels are influenced by patients’ lifestyle factors such as diet, exercise, and medication, achieving assay standardization is challenging [191,192]. Secondly, tumor heterogeneity has an important impact on tumor immunity in terms of lipid metabolism. For instance, the immune cells in the liver have a higher tolerance for lipid accumulation [126]. Therefore, DCs could maintain high immunogenic characteristics even under high lipid concentrations [125]. Thirdly, the studies comparing lipid metabolism indicators with traditional indicators are scarce. However, based on current research findings, the combined lipid metabolism indicators may improve diagnostic efficacy compared to current standards, such as Ki67 and clinical stage [189,190].

## 6. Tumor Therapeutic Strategies Targeting Lipid Metabolism Reprogramming

In this section, we initially describe the therapeutic strategy targeted at lipid metabolism molecules and the mitochondria. Then, we demonstrate the lipid metabolism reprogramming during chemotherapy and immunotherapy, and introduce a combination therapy as a potential solution. Upon review, the non-steroidal anti-inflammatory drugs (NSAIDs) and Chinese herbal medicine have exhibited a promising ability in combating tumors by reversing the abnormal lipid metabolism.

### 6.1. Targeting Lipid Metabolism Molecules, Mitochondria, and Lipid Metabolism Reprogramming-Related NcRNAs

The high-fat diet, also known as the Western diet, has been widely acknowledged as a risk factor for numerous cancers, including ESCC, GC, CRC, HCC, PDAC, BC, and PRAD [193,194,195]. Consequently, adopting a low-fat diet is a crucial strategy for preventing cancer [196]. Due to the abnormal lipid metabolism in tumor cells and the TME, the lipid metabolism inhibitors that target key molecules have emerged as a new therapeutic strategy. FASN inhibitors, including fasnall, TVB3166, AZ12756122, TVB-2640, and orlistat, have achieved preclinical success. In cervical cancer, TVB-2640 enhanced cisplatin sensitivity by promoting SLC7A11-mediated ferroptosis [197]. For BC, fasnall and TVB3166 decreased tumor cell proliferation by upregulating ceramide-induced apoptosis and endoplasmic reticulum stress [198,199]. The Akt/mTOR pathway, an important tumor-related pathway, could be inhibited by TVB3664 and AZ12756122, which ultimately suppressed the growth of HCC and NSCLC [200,201]. Orlistat suppressed peritoneal metastasis of ovarian cancer via inhibiting the AMPK/ACC signaling pathway-mediated energy supply [202]. Two clinical studies suggested the clinical feasibility of FASN inhibitors. One study found that the side effects of TVB-2640 on skin and eyes were non-serious and reversible, indicating its tolerable adverse reactions [203]. A prospective, single-center Phase II study in recurrent high-grade astrocytoma patients showed that combining TVB-2640 with bevacizumab improved 6-month progression-free survival, 6-month overall survival, and overall response rate to 31.4%, 68%, and 56%, respectively, compared to bevacizumab alone [204]. Darapladib, a lipoprotein-associated phospholipase A2 (Lp-PLA2) inhibitor, was initially developed to treat cardiovascular diseases [205]. In 2023, Oh et al. first discovered its application in tumor treatment as it could promote ferroptosis by preventing the cleavage of PE [206]. Furthermore, as Deng et al. suggested in an authentic review, monoacylglycerol lipase (MAGL) inhibitors can not only act as anti-cancer agents but also relieve cancer-associated symptoms such as pain, nausea, and vomiting [207]. However, it is essential to conduct clinical trials before these inhibitors can be utilized in clinical practice.

As the hub of lipid metabolism and cancer cell death, mitochondrion is a promising target for anti-tumor therapy [208]. For example, CPI-613 (Devimistat), a mitochondrial metabolism inhibitor, could activate AMPK and increase phosphorylation of ACC. This inhibits ACC activity and suppresses lipid metabolism, eventually promoting ROS-related apoptosis in PDAC cancer cells [209]. Matairesinol nanoparticles improve chemotherapy sensitivity to the FOLFOX regimen in CRC by inducing mitochondrial injury, oxidative damage, and TG reduction that results from inhibiting the expression of pancreatolipase (PNLIP) and DGAT2 [210]. In recent years, advancements in technology have led researchers to concentrate on mitochondrial functional modules that are involved in lipid metabolism and tumor cell death. Mitochondrial permeability transition pores (mPTPs), mitochondria-associated membranes (MAMs), and mitochondrial filament-forming protein LACTB are the representatives [211,212,213]. Adhering to the principle of “precision medicine”, targeting mitochondrial functional modules instead of the entire mitochondrion is the direction of future drug development. Since mitochondria are key organelles that maintain homeostasis in myocardial tissue; therefore, the cardiotoxicity of mitochondria-targeting drugs should be considered. Although Cascone et al. reckoned that cardiac injury mainly originates from mitotoxic effects, a recent review has established the status of mitochondrial damage [214,215]. Specifically speaking, the cardiotoxicity induced by anti-tumor drugs could be attributed to alteration of the mitochondrial respiratory chain, energy production, mitochondrial kinetics, and induction of mitochondrial oxidative/nitrosative stress [215]. Therefore, the toxicity of mitochondrial-targeting drugs should be addressed before being taken into clinical practice.

As we mentioned in Lipid Metabolism Reprogramming-Related Non-Coding RNAs (NcRNAs), ncRNAs including miR-15a-5p, miR-1275, LncRNA lnc030, LncRNA SLC25A21-AS1, and LncRNA ROLLCSC could mediate tumorigenesis by regulating the lipid metabolism of tumor cells. Therefore, targeting the above ncRNAs may become a new strategy for tumor therapy. However, few drugs were developed based on this strategy. For HCC, although genistein and vitamin D3 inhibited epithelial–mesenchymal transition, stemness, and proliferation by upregulating miR-1275 and miR-15a-5p, they were not used in lipid metabolism [216,217]. Considering the widespread distribution of ncRNAs, targeting ncRNAs deserves consideration for their non-target toxicity to normal cells. For instance, miRNA-15a-5p could induce arrhythmogenic right ventricular cardiomyopathy and myocardial fibrosis [218,219]. Similarly, miR-1275 aggravated cardiomyocyte injury, especially under ischemia-hypoxia, by secreting pro-inflammatory factors TNF-α and IL-1β, producing reactive oxygen species (ROS), and causing Ca2+ leakage from the sarcoplasmic reticulum [220,221,222]. Neuropsychiatric disorders, as common accompanying symptoms in tumor patients, can also be exacerbated by abnormal ncRNA levels. Numerous studies have proved that miR-15a-5p and miR-1275 could induce neuronal apoptosis, which eventually contributes to Parkinson’s disease, schizophrenia, and epilepsy [223,224,225]. Other adverse reactions, including intervertebral disc degeneration, keloid formation, and rheumatic autoimmune diseases, should not be overlooked either [226,227,228,229]. In conclusion, the above potential side effects become the main obstacle to the promotion of targeted ncRNA therapy. Therefore, developing ncRNA therapeutic drugs with cell specificity is the future direction.

### 6.2. Chemotherapy and Immunotherapy

During the chemotherapy, the lipid metabolism reprogramming is commonly observed, contributing to chemotherapy resistance [20]. Thus, targeting lipid metabolism has the potential to reverse this dilemma. In HCC, combining 4μ8C (endoplasmic reticulum emergency inhibitor) with the lipase inhibitor can reverse doxorubicin resistance caused by endoplasmic reticulum stress-induced lipid metabolism activation [230]. Cancer stem cells (CSCs), a distinct type of cancer cell, are the main cause of chemotherapy resistance. Furthermore, abnormal lipid metabolism can help sustain self-renewal, differentiation, invasion, metastasis, and drug resistance in CSCs [9]. Based on this, Liu et al. suggested the combination of Giripladib (an inhibitor of cytoplasmic phospholipase A2) with doxorubicin, cisplatin, or tamoxifen as a novel therapeutic strategy for CSCs elimination in BC. The underlying mechanism was attributed to the activation of the lncROPM-PLA2G16 pathway [231].

The relationship between lipid metabolism reprogramming and anti-tumor immunity is complicated, highlighting the necessity for combination therapy. On one hand, anti-tumor immunity influences lipid metabolism. As Zhao et al. observed, anti-PD-L1 therapy reduced resistance to cetuximab (an anti-EGFR treatment) by inhibiting the formation of the PD-L1/EGFR/ITGB4 triad, thereby reducing the accumulation of LDs, TGs, and CHO within HCC cells [55]. On the other hand, abnormal lipid metabolism impairs the anti-tumor immunity as well. In CRC, CYP19A1 abnormalized vascular, inhibited CD8^+^ T cell function, and reduced the efficacy of anti-PD-1 therapy by upregulating immune suppressors (PD-L1, IL-6, and TGF-β). Therefore, inhibiting the CYP19A1 may restore the effectiveness of anti-PD-1 therapy [232]. Targeting lipid metabolism reprogramming while activating anti-tumor immunity simultaneously may be the most effective treatment strategy and has achieved progress in nanomedicine. For instance, reduction-responsive RNAi nanoplatform could co-deliver monoacylglycerol lipase siRNA (siMGLL) and endocannabinoid receptor-2 siRNA (siCB-2) systemically, consequently suppressing the production of FFAs in pancreatic cancer cells and repolarizing TAMs into a tumor-inhibiting M1-like phenotype [233].

### 6.3. Non-Steroidal Anti-Inflammatory Drugs (NSAIDs)

Non-steroidal anti-inflammatory drugs (NSAIDs) are primarily utilized to treat fever, musculoskeletal pain, and neurodegenerative disease due to their excellent anti-inflammatory and analgesic properties [234,235]. In recent years, as researchers have discovered, apart from relieving the cancer-related pain, NSAIDs also played a vital role in anti-tumor activity, which was partly ascribed to the inhibition of cyclooxygenase (COX)-mediated phospholipid–arachidonic acid–prostaglandin E2 metabolism [236,237,238]. Notably, repressing lipid synthesis has also been identified as a significant anti-tumor mechanism. For instance, aspirin, one of the most representative NSAIDs, inhibited the lipid synthesis pathway involving c-myc, SREBP1, ACLY, ACC1, FASN, and SCD1. This inhibition helped suppress cancer cell growth and induced apoptosis in HER-2 positive BC [239]. Despite their potential to cause gastrointestinal toxicity to some extent, NASIDs remain a promising therapeutic strategy for non-cardia gastric and colorectal cancer [240,241].

### 6.4. Chinese Herbal Medicines

Chinese herbal medicines such as Astragalus membranaceus, Sophora flavescens, Cantharidin, Norcantharidin, and Berberine have been increasingly accepted by various countries as a strategy for treating digestive system tumors [242]. Numerous studies suggested that the primary mechanisms involve regulating anti-tumor immunity [243,244] and reprogramming lipid metabolism [245]. Ginseng-related metabolites are the representatives, as they could reverse the lipid metabolism disorder caused by tumorigenesis. For example, protopanaxadiol (PPD) regulated the lipid metabolism within CRC cells by inhibiting FASN expression and promoting the endoplasmic reticulum stress-induced cell death [246]. Similarly, ginsenoside compound K (GCK) exhibited excellent anti-CRC capacity through repressing the proliferation, migration, and invasion of cancer cells. The underlying mechanism was that GCK suppressed the protein expression of PLA2G16 and thereby reversed the lipid metabolism disorder [247]. The ginseng-related products mentioned above were derived from the gut bacterial metabolism, and intestinal microbial flora was a potential target for tumor therapy [248,249]. Thus, based on the low-fat diet, combining ginseng with intestinal flora-related therapy may offer a promising strategy for treating digestive tract tumors.

## 7. Conclusions

Lipid metabolism reprogramming plays a crucial role in cancer cells and immune cells within the TME, influencing tumorigenesis, diagnosis and prognosis, and therapeutic strategies. Targeting lipid metabolism molecules and mitochondria can help develop new treatment strategies for enhancing the effectiveness of chemotherapy and immunotherapy. Lipid metabolism-related molecules have significant potential in tumor diagnosis and prognosis, serving as new clinical indicators for cancer management. Moreover, NSAIDs and Chinese herbal medicines show promise in regulating lipid metabolism and anti-tumor immunity. Therefore, a deep understanding of the role of lipid metabolism reprogramming in tumors is essential for establishing more effective and precise diagnostic, monitoring, and treatment strategies.

Our review has the following advantages over prior literature. Firstly, unlike previous reviews that treated the lipid metabolism reprogramming of tumors as a whole, we elaborated on the changes in lipid metabolism of tumor cells and immune cells separately. Secondly, we illustrated the relationship between tumor-related genes and pathways and lipid metabolism. By targeting these molecules, cancer can be fought by reversing lipid metabolism reprogramming. Thirdly, we included the latest research results and expounded on the impact of ncRNAs on lipid metabolism in tumor cells. Last but not least, we introduced the role of traditional Chinese herbal medicines in targeted tumor lipid metabolism reprogramming.

Although our review has the above advantages, several unsolved problems should not be ignored. Systemic lipid levels, which can be affected by diet and body fat, reshape the lipid content of the TME [250,251]. However, the metabolic reprogramming changes within immune cells have not been fully elucidated. Currently, there are several research studies on NKs. Fasting could activate NKs by mobilizing fatty acids to supply their energy needs [161]. In contrast, obesity can inhibit tumor immunity by suppressing mTOR-mediated glycolysis through excessive accumulation of FA [162,163]. HDL and LDL are important indicators of survival prognosis for NSCLC patients receiving immune checkpoint inhibitor treatment [252]. Moreover, further studies have revealed that lipid metabolism genes can predict the response to immunotherapy in prostate neoplasms, lung adenocarcinoma, HCC, and BC [253,254,255,256]. Since lipid metabolism molecules are also expressed in normal cells, developing cell-specific lipid metabolism therapies is necessary.

In summary, future research should further explore the role of lipid metabolism reprogramming across different types of tumors and conduct large-scale prospective clinical studies to validate the clinical utility of these findings.

## Figures and Tables

**Table 1:** Lipid metabolism reprogramming in cancer cells.

	Molecular	Tumor	Reference
FA uptake			
	FABP, CD36	BC	[28]
		CRC	[29]
		HCC	[30]
		Bladder cancer	[31]
FA oxidation			
	CPT1A	BC	[32]
		CRC	[29]
FA synthesis			
	FASN, SCD1	BC	[33]
		CRC	[34]
		HCC	[35,36,37]
		CESC	[38]
		THCA	[39]
CHOL synthesis			
	SREBP1, LXR, PPARα	BC	[33]
		CRC	[34]
		HCC	[35,36,37]
Steroid synthesis			
	CYP17, CYP11A, CYP11B, βHSD	GC	[40]
		BC	[41,42,43,44]
		CRC	[45]
		Melanoma	[41]
		Prostate cancer	[41,46,47,48,49]
		Neuroblastoma	[50]
		Adrenal cortical tumor	[51]
Cancer genes			
	P53	Prostate cancer	[52]
	P63	SCC	[53]
	PI3k/AKT pathway	GC	[54]
		HCC	[55]
	MAPK pathway	HCC	[35]
		SCLC	[56]
		Lung cancer	[57]
		Pancreatic cancer	[58]
NcRNAs			
	MiR-15a-5p	Lung cancer	[59]
	MiR-1275	GC	[60]
	LncRNA lnc030	BC	[61]
	LncRNA SLC25A21-AS1	ESCC	[62]
	LncRNA ROLLCSC	Lung cancer	[63]

Abbreviations: FA, fatty acid; CRC, colorectal cancer; HCC, hepatoma carcinoma; BC, breast cancer; CPT1A, carnitine palmitoyl-transferase1A; FASN, fatty acid synthase; SCD1, stearoyl-CoA desaturase 1; CESC, cervical squamous cell carcinoma and endocervical adenocarcinoma; THCA, thyroid carcinoma; CHOL, cholesterol; SREBP1, sterol regulatory element-binding proteins 1; PPARα, peroxisome proliferator-activated receptor α; PRAD, prostate adenocarcinoma; SCC, squamous cell carcinoma; GC, gastric cancer; MAPK, mitogen-activated protein kinase, SCLC, small-cell lung cancer; ESCC, esophageal squamous cell carcinoma; CYP11A1, cytochrome P450 family 11 subfamily A member 1; CYP11B1, cytochrome P450 family 11 subfamily B member 1; CTP11B2, cytochrome P450 family 11 subfamily B member 2; CYP17, cytochrome P450 family 17; βHSD, b-hydroxysteroid dehydrogenase; NcRNAs, non-coding RNAs.

**Table 2:** Lipid metabolism of immune cells in the tumor microenvironment.

Immune Cells	Molecule	Influence of Lipid Metabolism on Immune Cells	Tumor	Reference
DCs				
	Lipids	Stimulate T cells	Lymphoma	[97]
		Build an immunosuppressive TME	Melanoma	[98]
		Antigen presentation, migration, and immune cell activation	MESA	[99]
T Cells				
	CHOL	TCR signal transduction	Melanoma, lung cancer, CRC	[100,101]
	SCD	Interactions among T cells	Melanoma, CRC, BC	[102]
	LA	Anti-tumor function	Neuroblastoma, lymphoma, melanoma, BC, CRC	[103]
	cPLA2, 5-LOX, LCAC	T cell exhaustion and functional defects	HCC	[104,105]
TAMs				
	LDs, CHOL	Differentiate into lipid-overburdened macrophages	BC, HCC, GBM, pancreatic cancer	[106,107,108,109,110,111]
TANs				
	Acrolein	Differentiate into TAN2	Glioma	[112]
	ASAH1	The formation of NETs	GBM	[113]
	ECL2	The NETs formation and the TANs recruitment	CRC	[114]
	CHOL	Interaction between TANs and NKs	CRC	[115]
	TGs	Interaction between TANs and cancer cells	Lung cancer	[116]
	PAF	Interaction between cancer cells and TANs	CRC, PDAC, lung cancer, ovarian cancer	[117]
NKs				
	Lipids	Anti-tumor function	CRC, HCC, PRAD, melanoma, oral cancer	[118,119,120]

Abbreviations: DCs, dendritic cells; TAMs, tumor-associated macrophages; TANs, tumor-associated neutrophils; NKs, natural killer cells; TME, tumor microenvironment; MESA, mesothelioma; TCR, T cell receptor; SCD, stearoyl-CoA desaturase; LA, linoleic acid; LCAC, long-chain acylcarnitine; cPLA2, c-phospholipase A2; 5-LOX, 5-lysyl oxidase; BC, breast cancer; HCC, hepatoma carcinoma; GBM, glioblastoma; LLMs, lipid-laden macrophages; TAFs, tumor-associated foam cells; OSMs, obesity-specific macrophages; LDLMs, lipid droplet-laden macrophages; LDs, lipid droplets; NETs, neutrophil extracellular traps; ASAH1, acyl sphingosine amidohydrolase 1; ECL2, enoyl-CoA δ-isomerase 2; TGs, triglycerides; PAF, platelet-activation factor; PDAC, pancreatic ductal adenocarcinoma; CHOL, cholesterol.

## Data Availability

Data are provided upon request for those interested.
